# Yttrium oxide nanoparticles induce potent selective cytotoxicity in HeLa cervical cance cells through ROS-mediated genomic instability and mitochondrial apoptosis

**DOI:** 10.1038/s41598-026-45693-w

**Published:** 2026-04-13

**Authors:** Hanan R. H. Mohamed, Shahd O. Elhaggan, Rawan S. Hekal, Chahd W. H. Fahmy, Zeina Noure, Nada Ahmed, Ayman Diab, Gehan Safwat

**Affiliations:** 1 Department of Zoology, Faculty of Science, Cairo University, Giza, Egypt; 2 Faculty of Biotechnology, October University for Modern Sciences and Arts (MSA), 6th of October City, Egypt

**Keywords:** Y_2_O_3_-NPs, Hela cervical cancer cells, Cytotoxicity, Oxidative stress, Genomic DNA integrity, Mitochondrial deplorization, Intrinsic apoptosis induction, Biochemistry, Cancer, Cell biology, Drug discovery, Molecular biology, Oncology

## Abstract

Cervical cancer remains a leading cause of cancer-related mortality, emphasizing the need for safer, more selective therapies. Yttrium oxide nanoparticles (Y_2_O_3_-NPs) have unique physicochemical properties, but their anticancer potential in cervical carcinoma remains underexplored. This study therefore estimated the cytotoxic effects of Y_2_O_3_-NPs in HeLa cervical cancer cells and normal HFB4 melanocytes, with mechanistic analyses focused on HeLa cells. Cells were exposed to Y₂O₃-NPs various concentrations and viability was assessed via MTT assay. Mechanistic endpoints; including total ROS generation, mitochondrial membrane potential, DNA damage, apoptotic morphology, and expression of apoptosis- and mitochondria-related genes, were analyzed using fluorescence assays, alkaline Comet assay, nuclear staining, and quantitative RT-PCR. Y_2_O_3_-NPs reduced HeLa cell viability in a concentration-dependent manner with markedly low IC50 value of 52.22 µg/mL (0.231 mM), whereas HFB4 cells were less affected and exhibited markedly greater IC50 value of 264.10 µg/mL (1.169 mM); high selectivity index of 5.06 demonstrating preferential cytotoxicity toward Hela cancer cells. Exposure to the IC50 concentration induced marked ROS overproduction, dramatic mitochondrial depolarization, severe DNA damage, and observable apoptotic nuclear changes in cancerous HeLa cells, accompanied by upregulation of apoptotic *p53,* anti-apoptotic *Bcl-2*, and mitochondrial *ND3* gene expression. Conclusion: These findings Y_2_O_3_-NPs exert strong and selective cytotoxic effects against HeLa cervical cancer cells while causing minimal toxicity to normal HFB4 melanocytes. This preferential cytotoxicity appears to be mediated by Y_2_O_3_-NPs–induced oxidative stress, genomic DNA damage, mitochondrial depolarization, and activation of mitochondrial-related apoptotic pathways. Although these results highlight the potential anticancer activity of Y_2_O_3_-NPs, further *in vivo* studies and detailed mechanistic investigations are needed to confirm their therapeutic efficacy and safety.

## Introduction

Cervical carcinoma remains a critical global health burden, ranking as the fourth most common malignancy among women. With over 600,000 new cases and 340,000 deaths reported annually, it is a leading cause of cancer-related mortality, particularly in low- and middle-income countries where screening and vaccination infrastructure are limited^1–3^. This disease primarily originates in the cervical transformation zone, a highly susceptible region where squamous and glandular columnar epithelia meet^4,5^. While histologically diverse, comprising squamous cell carcinoma (70–80%), adenocarcinoma, and rarer subtypes, nearly all cases share a singular etiological driver: persistent infection with high-risk human papillomavirus (HPV), specifically types 16 and 18^6,7^. With high-risk HPV detected in over 99% of cases, the virus is considered an indispensable factor in cervical carcinogenesis^8–11^. Despite advances in screening and HPV vaccination, cervical cancer still poses therapeutic challenges, particularly in advanced or treatment-resistant cases^12–14^. Current treatments; including surgery, chemoradiation, and systemic chemotherapy often lack cellular specificity, leading to off-target damage to healthy tissues. Consequently, HeLa cells have become a standard preclinical model for evaluating targeted therapies, including nanomaterials, radiosensitizers and redox-modulating compounds, due to their reliable representation of HPV-driven molecular dysfunction and high proliferative capacity^12–14^.

Among emerging nanotherapeutics, yttrium oxide nanoparticles (Y_2_O_3_-NPs) have gained attention for their unique physicochemical properties, including high thermal and chemical stability, excellent biocompatibility, versatile surface functionality, and notable redox-associated photonic characteristics^15–17^. These nanoparticles consequently can actively modulate the intracellular redox environment by generating reactive oxygen species (ROS), interacting with mitochondrial membranes, and triggering oxidative stress-mediated apoptosis^16–18^. While recent studies in breast, hepatic, and pancreatic cancer models have shown that Y_2_O_3_-NPs induce mitochondrial depolarization and genomic DNA damage, their effects have not yet been evaluated in cervical cancer models^16–19^. These cytotoxic mechanisms are particularly relevant to HeLa cells, which are characterized by dysregulated ROS homeostasis and *p53*-independent apoptotic signaling^20^.

A critical gap remains in understanding how Y_2_O_3_-NPs influence HeLa-specific oncogenic mechanisms, such *p53* inactivation, intrinsic ROS imbalance, genomic DNA damage, mitochondrial dysfunction, and apoptotic signals dysregulation. Furthermore, determining the therapeutic potential of Y_2_O_3_-NPs requires assessing their selectivity, specifically their impact on healthy human cells. Primary human melanocytes, which are highly sensitive to oxidative stress, serve as a rigorous model for evaluating biocompatibility and dermatologic safety^21,22^. Currently, no data exists regarding the impact of Y_2_O_3_-NPs on normal melanocytes. To address these gaps, this study aimed to preliminarily evaluate the cytotoxic effects of Y_2_O_3_-NPs in HeLa cervical cancer cells and normal human melanocytes (HFB4). In HeLa cells, we further investigated the nanoparticles’ potential to induce ROS-mediated genomic instability, mitochondrial depolarization, and apoptosis, providing initial mechanistic insights into their effects. These investigations are intended to generate foundational data for assessing the selective cytotoxic potential of Y_2_O_3_-NPs, while acknowledging that broader generalizations require additional studies in multiple cancer models.

## Materials and methods

### Chemicals and reagents

All chemicals and reagents used throughout the current study were of high analytical and molecular biology grade. Yttrium oxide nanoparticles (Y_2_O_3_-NPs) were obtained as a fine white powder from Sigma-Aldrich (St. Louis, MO, USA; Catalog No. 544892), with a reported average particle diameter of <50 nm and a purity of 99.9% (trace metals basis). To ensure experimental accuracy and consistency, all additional reagents and laboratory supplies were sourced at equivalent high-grade quality. Prior to experimental use, Y_2_O_3_-NPs were dispersed in dimethyl sulfoxide (DMSO; Sigma-Aldrich, USA) and sonicated for 15–20 min to achieve uniform suspension and reduce nanoparticle aggregation. Common laboratory reagents, including DMSO, 3-(4,5-dimethylthiazol-2-yl)-2,5-diphenyl tetrazolium bromide (MTT), and trypan blue dye, were purchased from Sigma-Aldrich (St. Louis, MO, USA).

Cell culture components; Dulbecco’s Modified Eagle Medium (DMEM), HEPES buffer, L-glutamine, gentamycin, and Trypsin–EDTA, were supplied by Lonza (Belgium). Growth media were supplemented with 10% fetal bovine serum (FBS) and 1% gentamycin. Phenol red–free media were used when required to eliminate spectrophotometric interference. All reagents were prepared immediately before use, and all experimental procedures were performed under sterile conditions in a Class II biological safety cabinet to ensure contamination-free handling and reproducible results.

### Characterization of the utilized nanoparticles

The Y_2_O_3_-NPs employed in the present study were previously characterized in detail by Emad *et al.*^16^ and Mohamed *et al.*^17^. Structural purity and crystalline phase composition of the purchased Y_2_O_3_-NPs were verified using X-ray diffraction analysis, which confirmed the absence of detectable impurities and the presence of the characteristic diffraction peaks corresponding to crystalline yttrium oxide. The colloidal stability and dispersion quality of the nanoparticle suspensions were further evaluated by dynamic light scattering, demonstrating uniform particle distribution and acceptable stability under experimental conditions.

Morphological characteristics of the Y_2_O_3_-NPs were examined using transmission electron microscopy (TEM). TEM micrographs revealed that the Y_2_O_3_-NPs were predominantly spherical in shape with well-defined boundaries and minimal aggregation. Quantitative image analysis indicated an average particle diameter of approximately 14.00 nm. These characterization results confirm the high purity, nanoscale dimensions, and favorable dispersion properties of the Y_2_O_3_-NPs, supporting their suitability for subsequent *in vitro* biological and mechanistic investigations^16,17^.

### Cultivation and maintaince of Hela cervical cancer cells and normal HFB4 melanocytes

Human cervical carcinoma HeLa cells and normal human HFB4 melanocytes were obtained from the Regional Center for Mycology and Biotechnology, Al-Azhar University, Cairo, Egypt. Upon receipt, cells were carefully revived and expanded under standard aseptic culture conditions to maintain optimal cell integrity and experimental reproducibility. HeLa cells were maintained in high-glucose DMEM (4.5 g/L) supplemented with 10% heat-inactivated FBS, 50 µg/mL gentamycin, 100 U/mL penicillin, and 100 µg/mL streptomycin to support the high metabolic demands of rapidly proliferating cancer cells. Normal HFB4 melanocytes were cultured in Melanocyte Growth Medium supplemented with 10% FBS and the same antibiotic regimen to maintain normal cell physiology. HFB4 cells were used within passages 5 –15 to ensure phenotypic stability and reproducibility. All cultures were incubated at 37°C in a humidified atmosphere with 5% CO₂, and culture media were replaced every 2–3 days to maintain nutrient availability and remove metabolic waste products. Cell morphology and confluence were monitored daily using an inverted phase-contrast microscope.

Subculturing was performed when cultures reached 70–80% confluence to avoid contact inhibition and phenotypic alterations. Cells were detached using 0.25% trypsin–EDTA, neutralized with complete growth medium, and reseeded at appropriate densities. Only cells in the exponential growth phase, exhibiting normal morphology and ≥90% viability (trypan blue exclusion), were used for all downstream experiments, including cytotoxicity, oxidative stress, genotoxicity, and apoptosis assays. Quality control measures were implemented to ensure the reliability of the results. HeLa and HFB4 cell lines were authenticated using short tandem repeat (STR) profiling prior to experiments. In addition, all cell cultures were routinely screened for mycoplasma contamination using the PCR-based MycoAlert™ Mycoplasma Detection Kit (Lonza). Testing was performed at four-week intervals throughout the study, and all cultures were consistently confirmed to be mycoplasma-free before and during experimentation.

### Screening the viability of human normal HFB4 melanocytes and Hela cervical cancer cells

The effect of Y_2_O_3_-NPs on the viability of normal human HFB4 melanocytes and human HeLa cervical cancer cells was evaluated using the colorimetric MTT assay, a well-established method for assessing mitochondrial metabolic activity as an indicator of cell viability, following previously reported protocols^23–25^. Cells were seeded into sterile 96-well flat-bottom culture plates (Falcon, NJ, USA) at a density of 1 × 10^4^ cells per well in 100 µL of complete DMEM containing 10% heat-inactivated FBS, 1% L-glutamine, 100 U/mL penicillin, and 100 µg/mL streptomycin. After 24 h of incubation under standard conditions (37 °C, 5% CO₂, humidified atmosphere) to allow cell attachment, the medium was carefully aspirated and replaced with treatment solutions. All experiments were conducted using cells between passages 5 and 15. Following cell attachment, the culture medium was carefully aspirated and replaced with fresh complete DMEM containing serial two-fold concentrations of Y_2_O_3_-NPs (7.8, 15.6, 31.25, 62.5, 125, 250, 500, and 1000 µg/mL). Each concentration was tested in triplicate wells, while untreated control wells received nanoparticle-free medium under identical conditions. Cells were then incubated with Y_2_O_3_-NPs for 72 h to allow sufficient interaction and cytotoxic response.

At the end of the exposure period, the treatment medium was removed and replaced with 100 µL of phenol red–free DMEM to prevent optical interference during absorbance measurement. Subsequently, 10 µL of MTT solution (12 mM stock; 5 mg/mL prepared in phosphate-buffered saline) was added to each well. Plates were incubated for an additional 4 h at 37°C in the dark, allowing metabolically active cells to reduce the MTT reagent into insoluble purple formazan crystals. After incubation, approximately 85 µL of the supernatant was gently aspirated from each well, and 50 µL of DMSO was added to solubilize the formed formazan crystals. Plates were incubated for a further 10 min at 37°C with gentle shaking to ensure complete dissolution. Absorbance was then recorded at 590 nm using a microplate reader (SunRise, TECAN, USA).

Cell viability was calculated as a percentage relative to untreated control cells using the following equation: Cell viability (%) = (OD^t^ / OD^c^) × 100; where OD^t^ represents the mean optical density of Y_2_O_3_-NPs–treated wells and OD^c^ corresponds to the mean optical density of control wells. The half-maximal inhibitory concentration (IC50) values were determined by nonlinear regression analysis using GraphPad Prism software (San Diego, CA, USA). All experiments were conducted independently at least three times, and data were expressed as mean ± standard deviation (SD). To evaluate the cancer cell–specific cytotoxicity of Y_2_O_3_-NPs, the Selectivity Index (SI) was calculated using the ratio of the IC50 value in normal HFB4 melanocytes to that in Hela cervical carcinoma cells, as follows: SI = (IC50 of normal HFB4/IC50 of Hela cancer cells).

### Treatment protocol for Hela cervical cancer cells

Human Hela cervical cancer cells were maintained in T25 culture flasks containing DMEM supplemented with 10% heat-inactivated FBS, 1% L-glutamine, and antibiotics (100 U/mL penicillin and 100 µg/mL streptomycin). Cells were incubated at 37°C in a humidified atmosphere with 5% CO_2_ and routinely monitored until reaching approximately 70–80% confluence, ensuring optimal growth conditions prior to treatment. At this stage, cells were randomly assigned into two experimental groups: an untreated control and Y_2_O_3_-NPs-treated groups. The control group received fresh culture medium containing <0.1% DMSO, while the treated group was exposed to Y_2_O_3_-NPs at the IC50 concentration previously determined via the MTT cytotoxicity assay. Both groups were incubated under identical conditions for 72 hours, allowing sufficient time for nanoparticle uptake and induction of cellular responses.

Following the treatment period, cells were harvested by detachment with 0.25% trypsin-EDTA, collected by centrifugation at 1,500 rpm for 5 minutes at 4°C, and washed twice with ice-cold phosphate-buffered saline (PBS, pH 7.4) to remove residual medium and unbound nanoparticles. The resulting cell pellets were carefully resuspended in PBS and stored at −80°C for downstream molecular and biochemical analyses, including gene expression and oxidative stress assays. All treatments were performed in triplicate to ensure reproducibility and enable robust statistical evaluation.

### Evaluation of genomic stability in HeLa cervical cancer cells

The genomic DNA stability and the potential genotoxic effect induced by Y_2_O_3_-NPs in human cervical carcinoma HeLa cells were assessed using the alkaline single-cell gel electrophoresis (comet) assay. This sensitive technique was performed in accordance with established and standardized protocols described by Tice *et al.*^26^ and Langie *et al.*^27^. HeLa cells were exposed to Y_2_O_3_-NPs at the previously determined IC50 concentration for 72 h prior to analysis. Following treatment, cells were harvested and approximately 1 × 10^4^ cells suspended in 15 µL of PBS were gently mixed with 60 µL of 0.5% low-melting-point agarose maintained at 37 °C. The cell-agarose mixture was immediately layered onto clean fully frosted microscope slides precoated with 1% normal-melting-point agarose. Slides were allowed to solidify at room temperature for 30 min.

Embedded cells were subsequently lysed by immersing the slides in freshly prepared cold lysis solution containing 2.5 M NaCl, 100 mM EDTA, and 10 mM Tris-HCl (pH 10), supplemented with 1% Triton X-100 and 10% DMSO. Lysis was carried out for 24 h at 4 °C in the dark to minimize additional oxidative or mechanical DNA damage. After lysis, slides were transferred to alkaline electrophoresis buffer (300 mM NaOH, 1 mM EDTA; pH 12) for 15 min to permit DNA unwinding and expression of alkali-labile sites. Electrophoresis was then conducted at 25 V and 300 mA for 30 min at 4 °C. Upon completion, slides were neutralized using 0.4 M Tris-HCl buffer (pH 7.5) for 5 min, fixed in chilled ethanol for an additional 5 min, and air-dried. DNA was stained with 50 µL of ethidium bromide solution (20 µg/mL) and visualized using a fluorescence microscope.

For each experimental group, fifty randomly selected nuclei per slide were captured and quantitatively analyzed using COMETSCORE™ image analysis software. DNA damage was evaluated using standard comet assay parameters, including tail length (extent of DNA migration), percentage of DNA in the tail, and tail moment (calculated as the product of tail length and tail DNA percentage divided by 100). All experiments were performed independently three times, and results were expressed as mean ± SD. Statistical analyses were applied to determine the significance of Y_2_O_3_-NPs–induced genotoxic effects in HeLa cervical cancer cells.

### Measurement of total ROS generation level in HeLa cervical cancer cells

The influence of a 72-h exposure of human cervical carcinoma HeLa cells to Y_2_O_3_-NPs at the predetermined IC50 concentration on total intracellular ROS generation was assessed using the fluorogenic probe 2′,7′-dichlorofluorescin diacetate (DCFH-DA). The assay was performed in accordance with the established method described by Siddiqui *et al.*^28^. Following treatment, equal volumes of HeLa cell suspension and DCFH-DA working solution (final concentration of 20 µM) were gently mixed in sterile microcentrifuge tubes. The mixtures were incubated for 30 min at room temperature in the dark to allow probe uptake. Once internalized, DCFH-DA was hydrolyzed by intracellular esterases to form the non-fluorescent compound dichlorofluorescin (DCFH), which was subsequently oxidized by intracellular ROS to generate the highly fluorescent dichlorofluorescein (DCF). The resulting fluorescence intensity directly reflected the intracellular ROS levels.

After incubation, stained cells were carefully mounted as thin monolayers onto clean, pre-labeled glass microscope slides. Fluorescent signals were visualized using an epifluorescence microscope equipped with appropriate excitation and emission filters for DCF detection. Images were captured at 200× magnification from randomly selected microscopic fields, while maintaining identical exposure parameters for all experimental groups to ensure accurate quantitative comparison. Quantitative analysis of fluorescence intensity was performed using *Fiji* (ImageJ) image analysis software. Relative total ROS generation levels were calculated by comparing Y_2_O_3_-NPs–treated cells with untreated control cells. All experiments were carried out in triplicate, and the data were used to ensure reproducibility and statistical reliability of the results.

### Fluorometric analysis of mitochondrial membrane potential integrity in HeLa cervical carcinoma cells

Alterations in mitochondrial membrane potential, a key indicator of mitochondrial integrity and an early event in apoptosis, were evaluated in human cervical carcinoma HeLa cells following exposure to Y_2_O_3_-NPs. Hela ells were treated with Y_2_O_3_-NPs at the previously established IC50 concentration for 72 h prior to analysis. Mitochondrial polarization status was assessed using Rhodamine-123, a lipophilic, cationic fluorescent probe that preferentially accumulates within functional, energized mitochondria, following a protocol adapted from Zhang *et al.*^29^. Briefly, equal volumes of HeLa cell suspension and Rhodamine-123 working solution (final concentration: 10 µg/mL) were gently mixed in light-protected sterile microcentrifuge tubes and incubated at 37 °C for 1 h in the dark to facilitate intracellular dye uptake. Following incubation, cells were washed twice with ice-cold PBS to remove excess dye and minimize nonspecific background fluorescence.

Stained cells were then evenly distributed onto clean glass microscope slides to form a uniform monolayer and covered with sterile coverslips. Fluorescence imaging was performed using an epifluorescence microscope equipped with appropriate filters for Rhodamine-123 detection. Images were captured at 200× magnification from randomly selected microscopic fields while maintaining identical exposure parameters across all experimental groups. Quantitative analysis of fluorescence intensity, indicative of mitochondrial membrane polarization, was carried out using *Fiji* (ImageJ) software. A marked reduction in Rhodamine-123 fluorescence intensity in Y_2_O_3_-NPs–treated HeLa cells compared with untreated controls was interpreted as mitochondrial membrane depolarization, reflecting mitochondrial dysfunction and early activation of apoptotic pathways. All experiments were conducted in triplicate, and results were expressed as mean ± SD to ensure reproducibility and statistical validity.

### Screening apoptotic induction in Hela cervical cancer cells

Apoptosis, the programmed cell death process critical for eliminating damaged or malignant cells, is a key indicator of anticancer efficacy. In the present study, the pro-apoptotic effects of Y_2_O_3_-NPs were assessed in human cervical carcinoma HeLa cells following a 72-hour exposure at the previously determined IC50 concentration. Untreated HeLa cells served as negative controls. To ensure comprehensive detection of apoptosis, two complementary approaches were employed: (*i*) the chromatin diffusion assay to detect DNA fragmentation, a hallmark of late-stage apoptosis, and (*ii*) nuclear staining with 4′,6-diamidino-2-phenylindole (DAPI) to visualize characteristic nuclear alterations, including chromatin condensation and fragmentation. All assays were performed in triplicate, and results were expressed as mean ± SD to ensure reproducibility and statistical reliability.

#### Chromatin diffusion assay

The chromatin diffusion assay identifies apoptotic cells based on the presence of alkali-labile sites within fragmented DNA, which diffuse under alkaline conditions to form a distinct halo surrounding the nucleus^30^. For the assay, slides were pre-coated with 0.7% normal-melting-point agarose. HeLa cells were embedded in 0.5% low-melting-point agarose and layered onto the coated slides. After air-drying, slides were subjected to cold lysis to remove cytoplasmic contents while preserving nuclear DNA. Slides were subsequently neutralized with Tris buffer, fixed in chilled ethanol, and stained with ethidium bromide. Apoptotic cells were identified by the presence of peripheral DNA halos under a fluorescence microscope. For each treatment group, 1,000 cells were analyzed to determine the percentage of cells exhibiting DNA fragmentation.

#### DAPI nuclear staining

DAPI, a DNA-specific fluorescent dye, was used to corroborate apoptotic induction by detecting chromatin condensation and nuclear fragmentation^31^. HeLa cells were seeded in 96-well plates at a density of 1 × 10^4^ cells per well and allowed to adhere overnight. Following exposure to Y_2_O_3_-NPs at the IC50 concentration for 72 h, cells were gently washed with PBS, fixed with 4% paraformaldehyde for 15 min at room temperature, and stained with DAPI (1 µg/mL in PBS) for 1 h in the dark. Fluorescence imaging was performed using an epifluorescence microscope equipped with appropriate filters at 200× magnification. Apoptotic nuclei were distinguished by their bright, condensed, and fragmented appearance, whereas non-apoptotic nuclei displayed uniform, diffuse fluorescence. A total of 1,000 nuclei per experimental group were evaluated to quantify the percentage of apoptotic cells.

### Quantitative analysis of apoptosis- and mitochondria-related gene expression in HeLa cervical cancer cells

To investigate the molecular effects of Y_2_O_3_-NPs on mitochondrial function and apoptotic pathways in HeLa cervical cancer cells, quantitative real-time polymerase chain reaction (qRT-PCR) was employed to assess the mRNA levels of the pro-apoptotic gene *p53*, the mitochondrial *ND3* gene (a key component of the electron transport chain), and the anti-apoptotic gene *Bcl-2*. HeLa cells, both untreated and treated with Y_2_O_3_-NPs at the previously determined IC50 concentration for 72 h, were harvested for total RNA extraction using the GeneJET RNA Purification Kit (Thermo Fisher Scientific, USA) according to the manufacturer’s instructions. RNA yield and purity were verified using a NanoDrop spectrophotometer. One microgram of purified RNA from each sample was reverse-transcribed into complementary DNA (cDNA) using the High-Capacity cDNA Reverse Transcription Kit (Applied Biosystems, USA). qRT-PCR amplification was performed using SYBR Green PCR Master Mix and gene-specific primers listed in Table 1^32–34^ on a StepOnePlus Real-Time PCR System (Applied Biosystems, USA). GAPDH was used as the endogenous control to normalize gene expression. Relative expression levels of *p53, ND3,* and *Bcl-2* were calculated using the comparative Ct (2^-ΔΔCt^) method. All reactions were conducted in triplicate to ensure accuracy and reproducibility, and the data were presented as mean ± standard deviation (SD).

**Table 1:** Sequences of primers used in qRT-PCR.

Gene	Strand	Primer’s sequences
GAPDH	Forward	5’-GAAGGTGAAGGTCGGAGTCA-3’
	Reverse	5’-GAAGATGGTGATGGGATTTC-3’
ND3	Forward	5’-CGCCGCCTGATACTGGCAT-3’
	Reverse	5’-CTAGTATTCCTAGAAGTGAG-3’
BCL-2	Forward	5’-TCCGATCAGGAAGGCTAGAGT-3’
	Reverse	5’-TCGGTCTCCTAAAAGCAGGC-3’
P53	Forward	5’-CAGCCAAGTCTGTGACTTGCACGTAC-3’
	Reverse	5’-CTATGTCGAAAAGTGTTTCTGTCATC-3’

### Statistical analysis

All experimental data were analyzed using the Statistical Package for the Social Sciences (SPSS, Version 21, IBM, USA). Results from the alkaline comet assay, qRT-PCR, mitochondrial membrane potential assessment, ROS quantification, and apoptosis assays are presented as mean ± SD. Each experiment included at least three independent biological replicates, with a minimum of three technical replicates per condition, as specified in the figure legends. Comparisons between Y_2_O_3_-NPs-treated and control Hela cells were performed using an unpaired two-tailed Student’s t-test for pairwise comparisons, after verifying normality and homogeneity of variance. Individual data points are displayed where feasible to illustrate variability. Differences were considered statistically significant at p < 0.05.

## Results

### Yttrium oxide nanoparticles exhibit potent and preferential cytotoxicity on Hela cervical cancer cells

**Fig. 1:**
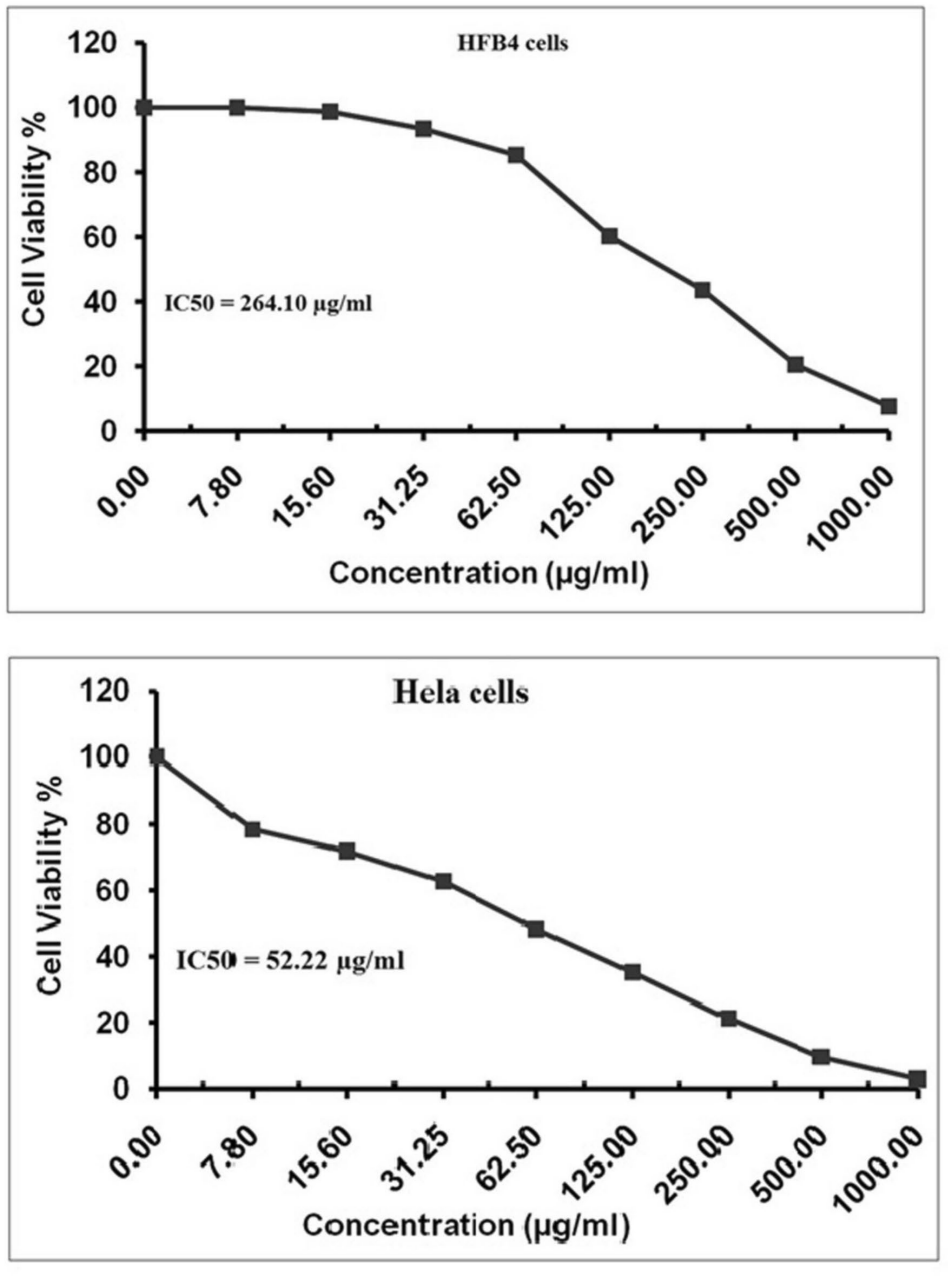
Viability of HeLa cervical cancer cells and normal human HFB4 melanocytes determined by the MTT assay after 72 h exposure to Y_2_O_3_-NPs at two-fold increasing concentrations (7.8–1000 µg/mL). Data are presented as mean ± SD from three independent biological experiments, each performed in triplicate.

Exposure of HeLa cells to Y_2_O_3_-NPs various concentrations for 72 hours caused a pronounced, concentration-dependent decrease in HeLa cell viability (Fig. 1). HeLa cells exhibited a markedly low IC50 value of 52.22 µg/mL (0.231 mM), indicating strong sensitivity to Y_2_O_3_-NPs toxicity and potent antiproliferative activity. In contrast, normal HFB4 melanocytes were considerably less affected, with only modest viability reduction at the highest Y_2_O_3_-NPs concentrations and a substantially higher IC50 value of 264.10 µg/mL (1.169 mM) as seen in Fig. 1. This difference in cytotoxicity resulted in a high selectivity index of 5.06, demonstrating that Y_2_O_3_-NPs preferentially target HeLa cervical cancer cells while largely sparing normal melanocytes. Given this pronounced selectivity and potency, subsequent analyses focused on the molecular mechanisms underlying Y_2_O_3_-NPs –induced cytotoxicity in HeLa cells.

### Yttrium oxide nanoparticles elicit severe genomic instability in HeLa cervical cancer cells

**Fig. 2:**
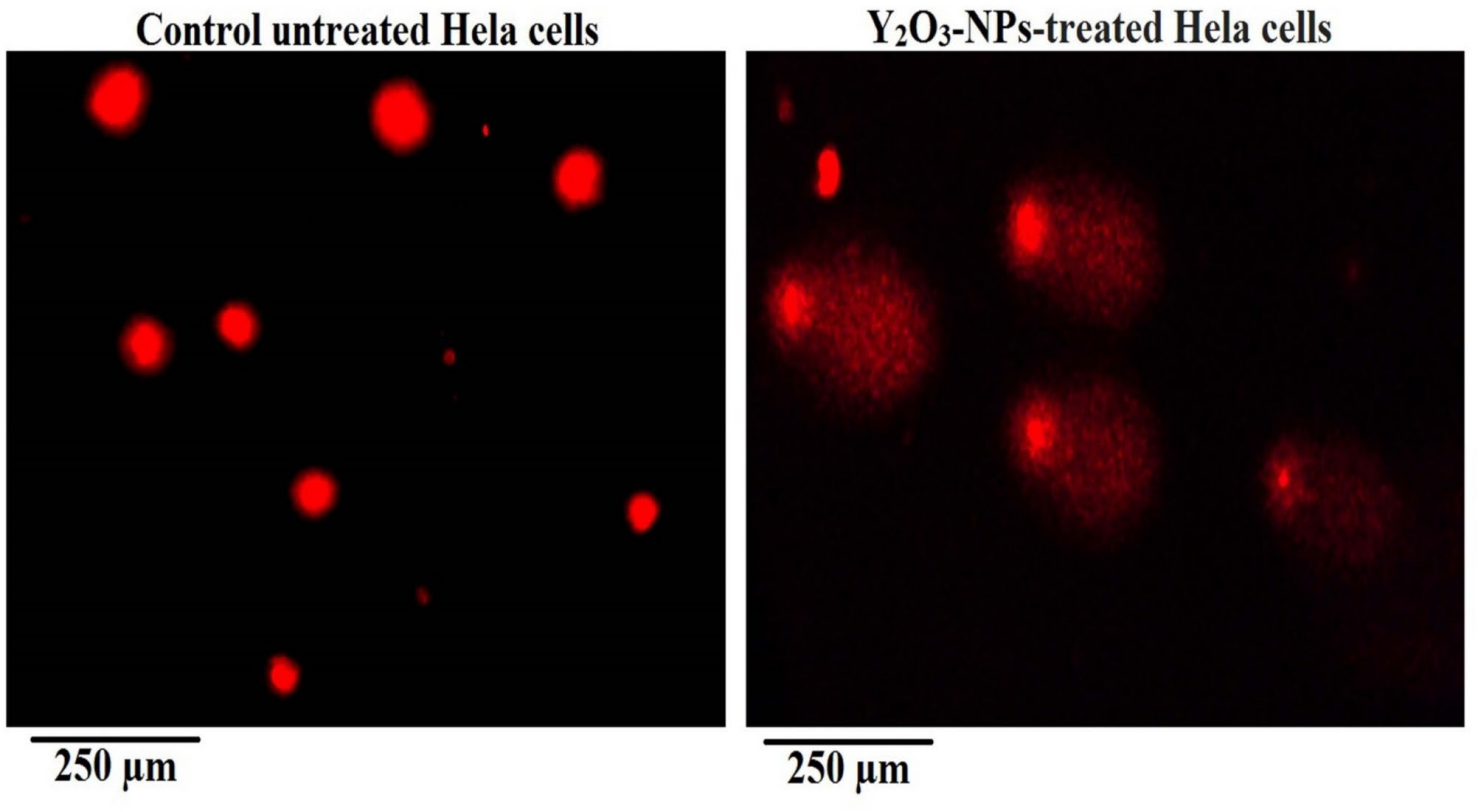
Representative comet assay images showing nuclei with intact DNA in untreated HeLa cells and nuclei with DNA damage in cells treated with Y_2_O_3_-NPs at the IC50 concentration (52.22 µg/mL) for 72 h. Quantitative comet parameters were calculated from 50 cells per sample across three independent experiments. Images are representative. Magnification: 200×.

Comet assay results showed that exposure of HeLa cells to Y_2_O_3_-NPs at the IC50 concentration of 52.22 µg/mL (0.231 mM) for 72 h significantly increased DNA damage compared with untreated control Hela cells (Table 2, Fig. 2). Quantitatively, measured Comet parameters: tail length, %DNA in tail, and tail moment were all significantly elevated (p < 0.001) in Y_2_O_3_-NPs -treated Hela cells (Table 2), indicating increased DNA strand breaks and alkali-labile lesions following Y_2_O_3_-NPs exposure. Y_2_O_3_-NPs –exposed cells showed pronounced DNA migration from the nuclear head into the tail region, whereas control cells maintained predominantly compact nuclei with minimal DNA migration. Representative micrographs (Fig. 2) aligned with the quantitative data, revealing distinct comet structures in Y_2_O_3_-NPs –treated Hela cells and mostly intact nuclei in control cells. Collectively, these findings confirm a significant elevation of genomic DNA damage in HeLa cells after Y_2_O_3_-NPs exposure and indicate that genotoxic stress is associated with the observed reduction in cell viability.

**Table 2:** Induction level of genomic DNA damage in human Hela cervical cancer cells detected using alkaline comet assay after 72 h exposure to the IC50 concentration (52.22 µg/mL) of Y_2_O_3_-NPs.

	Treatment (Concentration)	Tail length (px)	%DNA in tail	Tail moment
Hela cervical cancer cells	Untreated (0.00 µg/ml/mM)	1.76 ± 0.10	15.43 ± 1.80	0.29 ± 0.02
	Y_2_O_3_-NPs-treated (52.22 µg/mL= 0.231 mM)	18.26 ± 1.08^***^	38.86 ± 1.72^***^	6.99 ± 0.50^***^

Results are expressed as mean ± SD.

^***^: Indicates statistical significant difference from the compared untreated control cells at p<0.001, using *independent student t-test.*

### Yttrium oxide nanoparticles induce marked total intracellular ROS production in HeLa cervical cancer cells

**Fig. 3:**
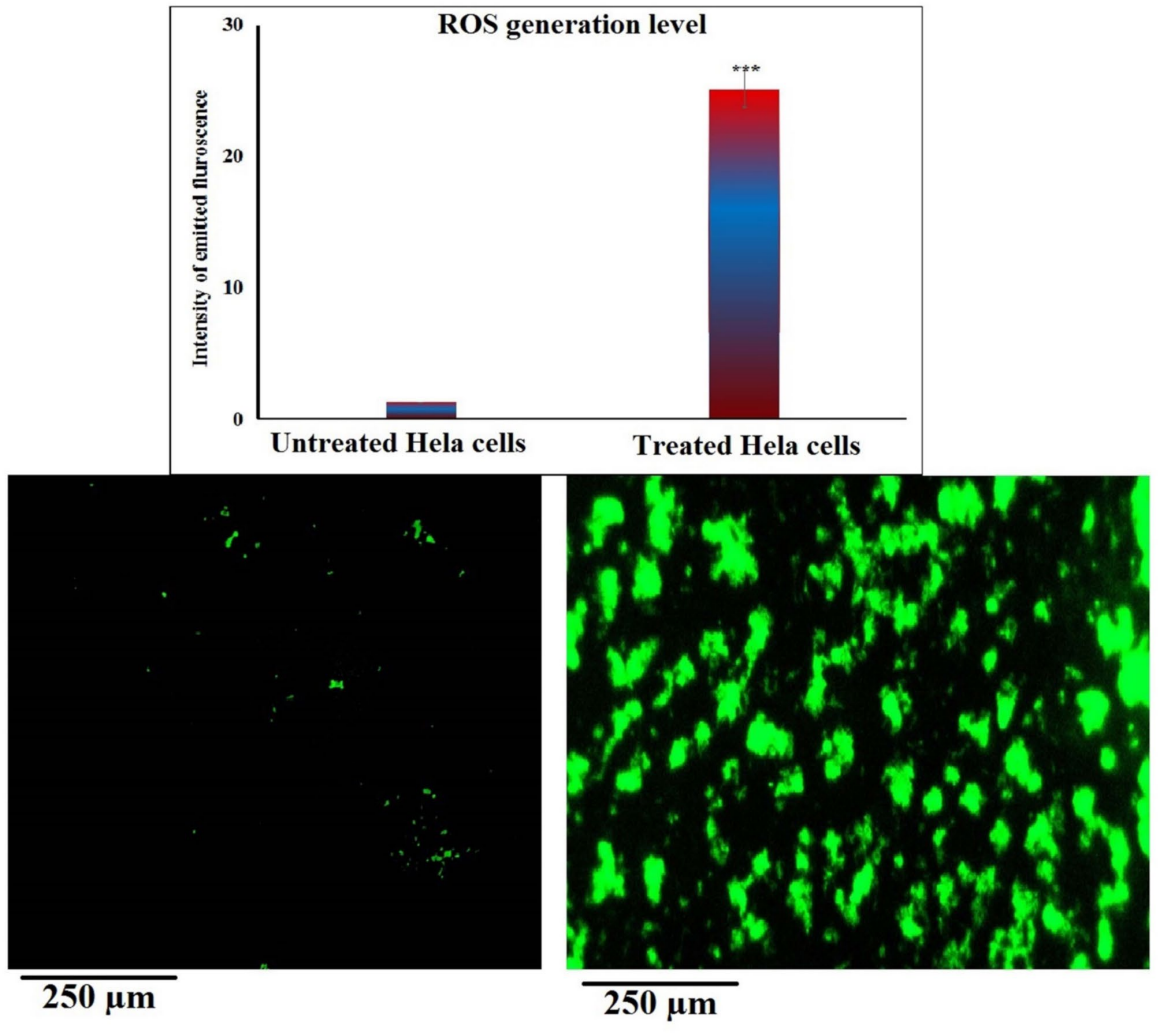
Intracellular ROS levels in untreated control and Y_2_O_3_-NPs–treated HeLa cells (52.22 µg/mL, 72 h) assessed using DCFH-DA. Representative fluorescence images (200×) are shown alongside quantitative analysis. Quantification was based on measurements from multiple fields per sample in three independent experiments. Data are expressed as mean ± SD.

Exposure of HeLa cervical cancer cells to Y_2_O_3_-NPs at the IC50 concentration of 52.22 µg/mL (0.231 mM) for 72 h resulted in a significant increase (p < 0.001) in total intracellular ROS generation compared with untreated control Hela cells (Fig. 3). Quantitative fluorescence measurements showed a marked elevation in total ROS production level in Y_2_O_3_-NPs -treated Hela cells, while control cells maintained low basal signals. Representative images in Fig. 3 are consistent with the quantitative data, showing that the treated cells exhibited stronger fluorescence intensity than the control cells. These findings indicate that Y_2_O_3_-NPs exposure is associated with a significant rise in intracellular ROS generation in HeLa cervical cancer cells, reflecting oxidative stress that may contribute to the observed cytotoxic effects.

### Yttrium oxide nanoparticles cause profound mitochondrial membrane depolarization in HeLa cervical cancer cells

**Fig. 4:**
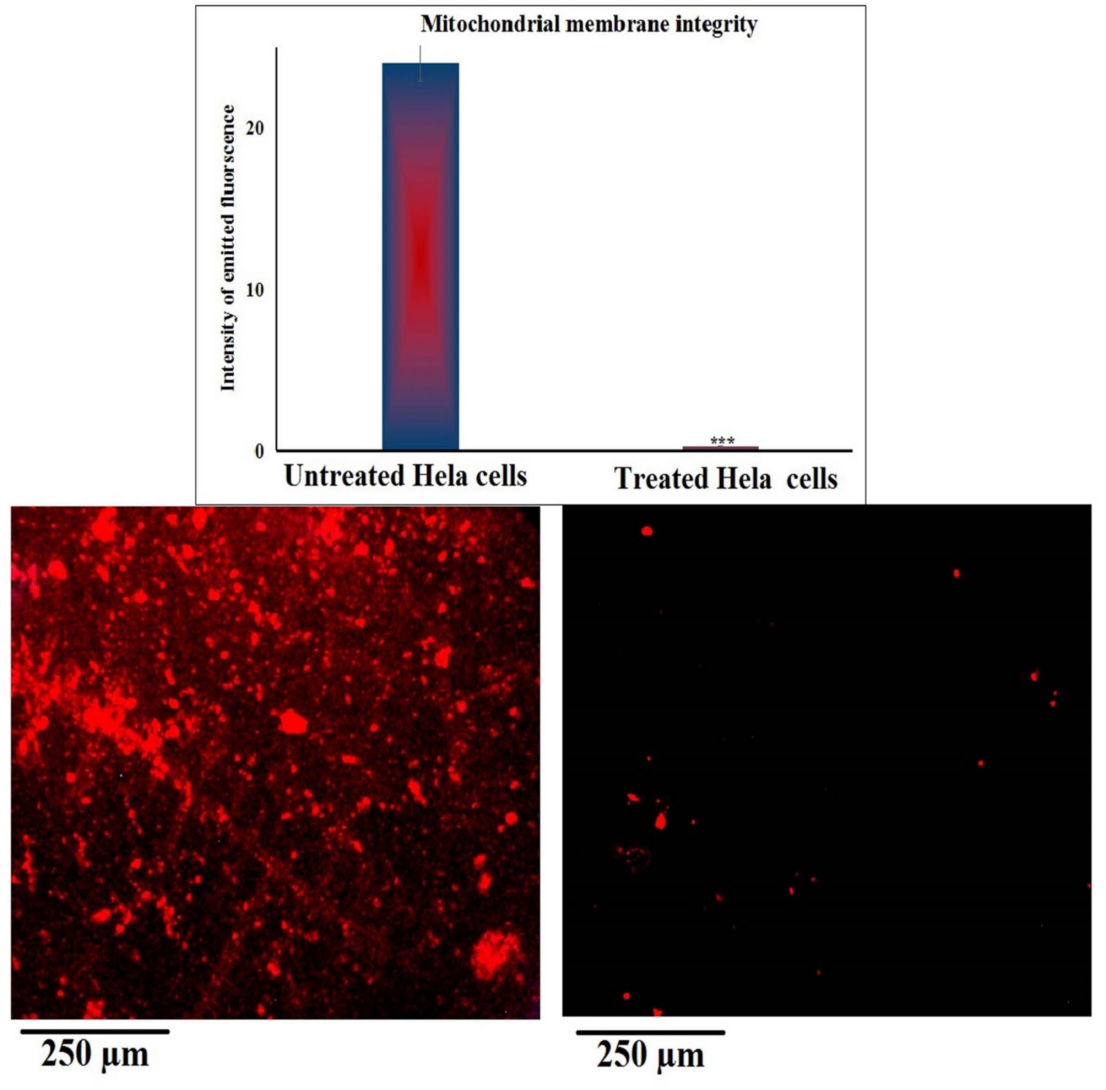
Mitochondrial membrane potential in untreated control and Y_2_O_3_-NPs–treated HeLa cells (52.22 µg/mL, 72 h) evaluated using Rhodamine-123 staining. Representative fluorescence images (200×) and corresponding quantitative analysis are shown. Quantification included multiple fields per condition across three independent experiments.

As depicted in Fig. 4, HeLa cells exposed to Y_2_O_3_-NPs at the IC50 concentration of 52.22 µg/mL (0.231 mM) for 72 h exhibited a significant reduction in mitochondrial membrane potential compared with untreated control Hela cells. Quantitative analysis revealed a highly significant decrease in Rhodamine-123 fluorescence intensity in Y_2_O_3_-NPs-treated Hela cells (p < 0.001), indicating substantial mitochondrial depolarization. This decline reflects a loss of mitochondrial electrochemical gradient and impaired mitochondrial integrity in Y_2_O_3_-NPs–exposed Hela cells. Consistent with these measurements, representative fluorescence images in Fig. 4 revealed weaker and less defined mitochondrial staining in Y_2_O_3_-NPs-exposed Hela cells, whereas untreated control cells maintained strong and uniform fluorescence. Collectively, these findings demonstrate that Y_2_O_3_-NPs exposure leads to a pronounced reduction in mitochondrial membrane potential in HeLa cells, reflecting significant mitochondrial dysfunction that coincides with the observed cytotoxicity.

### Yttrium oxide nanoparticles induce pronounced apoptotic cell death in HeLa cervical cancer cells

**Fig. 5:**
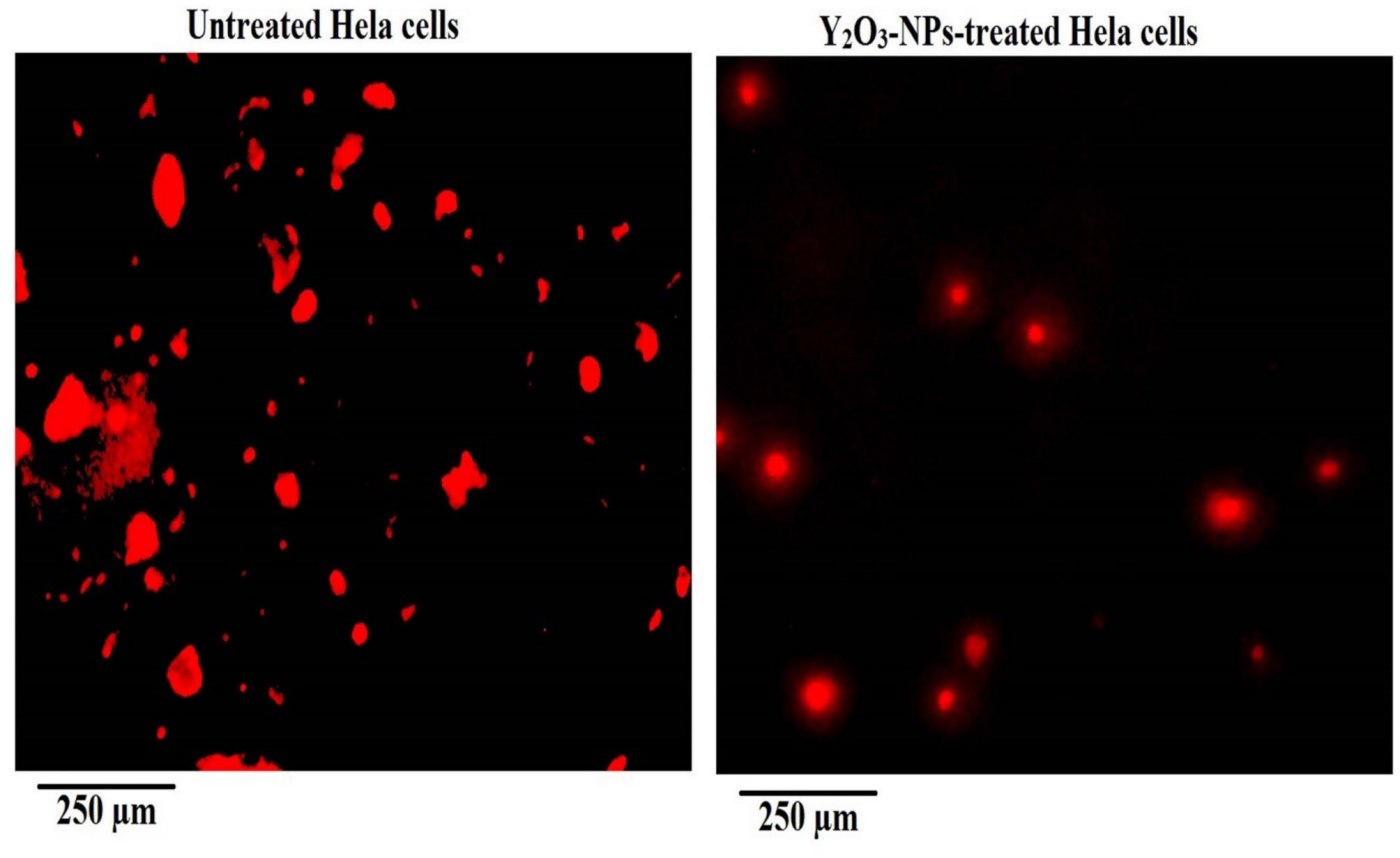
Chromatin diffusion assay showing normal nuclei with intact DNA in control cells and apoptotic nuclei with diffused DNA in HeLa cells treated with Y_2_O_3_-NPs (52.22 µg/mL, 72 h). Apoptotic indices were quantified from multiple fields per sample in three independent experiments. Images are representative. Magnification: 200×.

Apoptosis analysis revealed a significant increase in programmed cell death in HeLa cells after 72 h exposure to Y_2_O_3_-NPs at the IC50 concentration of 52.22 µg/mL (0.231 mM). Quantitative analysis demonstrated a highly significant increase in the proportion of apoptotic Hela cells compared with untreated control cells (p < 0.001) following Y_2_O_3_-NPs exposure. In the chromatin diffusion assay, Y_2_O_3_-NPs–treated cells showed a marked rise in halo-positive nuclei, indicative of extensive DNA fragmentation, whereas control cells maintained compact, intact nuclei with negligible halo formation as displayed in Table 3 and Fig. 5. The percentage of apoptotic nuclei increased dramatically in treated cultures, confirming that Y_2_O_3_-NPs strongly promote DNA disintegration in HeLa cervical cancer cells.

**Table 3:** Incidence of apoptosis induction in human Hela cervical cancer cells estimated using chromatin diffusion assay after 72 h exposure to the IC50 concentration (52.22 µg/mL) of Y_2_O_3_-NPs.

		Number of cells with		Percentage of apoptotic cells
	Treatment (Concentration)	Intact DNA	Diffused DNA	
Hela cervical cancer cells	Untreated (0.00 µg/ml/mM)	967.00 ± 9.54	33.00 ± 9.54	3.30 ± 0.95
	Y_2_O_3_-NPs-treated (52.22 µg/mL= 0.231 mM)	134.33 ± 10.07^***^	865.67 ± 10.07^***^	86.57 ± 1.07^***^

Results are expressed as mean ± SD.

^***^: Indicates statistical significant difference from the compared untreated control cells at p<0.001, using *independent student t-test.*

**Fig. 6:**
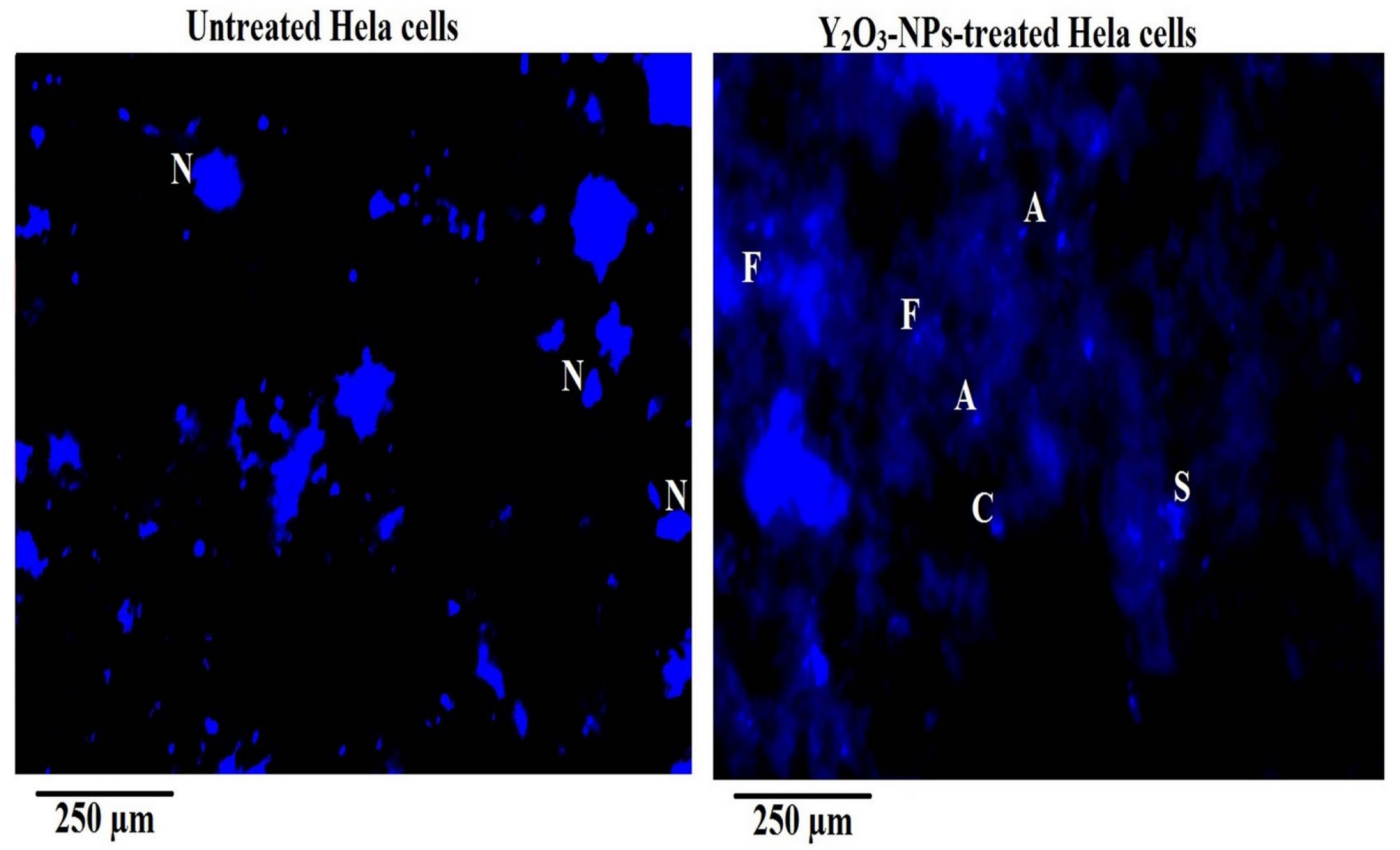
DAPI staining of HeLa cells showing intact nuclei in untreated control cells and condensed/fragmented nuclei in cells treated with Y_2_O_3_-NPs (52.22 µg/mL) for 72 h. Apoptotic nuclei were quantified from multiple fields across three independent experiments. N: Normal nuclei; S: Nuclear shrinkage; C: Chromatin condensation; F: Nuclear fragmentation and A: Apoptotic body. Magnification: 200×.

DAPI nuclear staining further corroborated these findings. Untreated HeLa cells exhibited normal round and uniformly stained nuclei, whereas Y_2_O_3_-NPs–exposed cells displayed classical apoptotic features, including intense chromatin condensation, nuclear shrinkage, nuclear fragmentation, and formation of apoptotic bodies as seen in Fig. 6. Quantitative scoring revealed a significant elevation (p < 0.001) in the number and percentage of Hela cells exhibiting these nuclear abnormalities in treated samples compared with untreated control Hela cells (Table 4). Together, the consistency across independent apoptosis assays confirms that Y_2_O_3_-NPs effectively induce apoptosis in HeLa cervical cancer cells. Pronounced nuclear condensation, chromatin fragmentation, and apoptotic body formation observed in Y_2_O_3_-NPs-treated Hela cells indicate activation of intrinsic apoptotic pathways, likely driven by Y_2_O_3_-NPs–mediated oxidative stress, mitochondrial depolarization, and DNA damage. These results strongly support apoptosis as a central mechanism underlying the selective cytotoxicity of Y_2_O_3_-NPs, consistent with the observed reduction in cell viability.

**Table 4:** Incidence of apoptosis induction in human Hela cervical cancer cells assessed by DAPI staining after 72 h exposure to the IC50 concentration (52.22 µg/mL) of Y_2_O_3_-NPs.

		Number of cells with		Percentage of apoptotic cells
	Treatment (Concentration)	Intact DNA	Fragmented and condensed DNA	
Hela cervical cancer cells	Untreated (0.00 µg/ml/mM)	965.00 ± 6.08	35.00 ± 6.08	3.50 ± 0.61
	Y_2_O_3_-NPs-treated (52.22 µg/mL= 0.231 mM)	91.00 ± 6.56^***^	909.00 ± 6.56^***^	90.90 ± 0.65^***^

Results are expressed as mean ± SD.

^***^: Indicates statistical significant difference from the compared untreated control cells at p<0.001, using *independent student t-test.*

### Yttrium oxide nanoparticles profoundly disrupt apoptotic *p53*, mitochondrial *ND3*, and anti-apoptotic *Bcl-2* gene expression in HeLa cervical cancer cells

qRT-PCR analysis demonstrated that exposure of HeLa cervical cancer cells to Y_2_O_3_-NPs at the IC50 concentration of 52.22 µg/mL (0.231 mM) for 72 hours induced significant alterations in the expression of genes critically involved in apoptosis and mitochondrial function regulation (Table 5). Specifically, the pro-apoptotic tumor suppressor *p53* was markedly upregulated (p < 0.001) in Y_2_O_3_-NPs-treated Hela cells compared with untreated control cells, indicating activation of intrinsic apoptotic pathways in response to Y_2_O_3_-NPs–induced cellular stress. The increase in *p53* expression likely reflects a cellular response to DNA damage and oxidative stress, promoting the elimination of damaged or stressed cells through programmed cell death. In parallel, the mitochondrial *ND3* gene, a core subunit of complex I of the electron transport chain, was significantly upregulated (p < 0.001), suggesting dramatic disruption of mitochondrial bioenergetics and enhanced mitochondrial stress. Such marked upregulation aligns with the observed loss of mitochondrial membrane potential and supports a mechanism in which mitochondrial dysfunction contributes to apoptosis induction.

**Table 5:** Fold change in the expression level of *p53, ND3* and *Bcl2* genes in human Hela cervical cancer cells after 72 h exposure to the IC50 concentration (52.22 µg/mL) of Y_2_O_3_-NPs.

	Treatment (Concentration)	*p53*	*ND3*		*Bcl2*
Hela cervical cancer cells	Untreated (0.00 µg/ml/mM)	1.00 ± 0.00		1.00 ± 0.00	1.00 ± 0.00
	Y_2_O_3_-NPs-treated (52.22 µg/mL= 0.231 mM)	9.36 ± 0.23^***^		3.20 ± 0.10^***^	5.37 ± 0.17^***^

Results are expressed as mean ± SD.

^***^: Indicates statistical significant difference from the compared untreated control cells at p<0.001, using *independent student t-test.*

Similarly, the anti-apoptotic *Bcl-2* gene expression was also significantly elevated (p < 0.001) in Y_2_O_3_-NPs-exposed Hela cancer cells compared to untreated control cells (Table 5). Collectively, the simultaneous marked upregulation of *p53, ND3*, and *Bcl-2* underscores a complex, multifaceted cellular response to Y_2_O_3_-NPs in HeLa cervical carcinoma cells. These transcriptional alterations reveal that Y_2_O_3_-NPs induce apoptosis in Hela cervical cancer cells while disrupting mitochondrial homeostasis. This dual effect highlights the potent capacity of Y_2_O_3_-NPs to target HeLa cells selectively, providing mechanistic insight into their potential application as nanotherapeutic agents for cervical cancer treatment.

## Discussion

The aggressive biological nature of cervical carcinoma, together with its high global prevalence, particularly in low- and middle-income countries, continues to pose a major clinical challenge. Current treatment modalities, especially chemotherapy, suffer from several critical limitations, including low tumor specificity, the frequent development of chemoresistance, and severe systemic toxicities such as nephrotoxicity, myelosuppression, neurotoxicity, and gastrointestinal complications. Collectively, these adverse effects substantially restrict the long-term efficacy of chemotherapy and negatively impact patient quality of life^35,36^. These challenges underscore the urgent need for novel, selective, and effective therapeutic approaches capable of overcoming the limitations of conventional treatments and improving clinical outcomes. Nanotechnology offers promising solutions in cancer therapy by enabling targeted exploitation of tumor-specific vulnerabilities and promoting oxidative stress–mediated cancer cell death^37,38^. In this context, Y_2_O_3_-NPs exhibit distinctive physicochemical and redox properties that may be advantageous for anticancer applications. However, their therapeutic potential in cervical carcinoma remains fully unexplored, since previous investigations have focused predominantly on non-cervical cancer models including breast, epidermoid skin, pancreatic and liver cancer^16–19^.

**Fig. 7:**
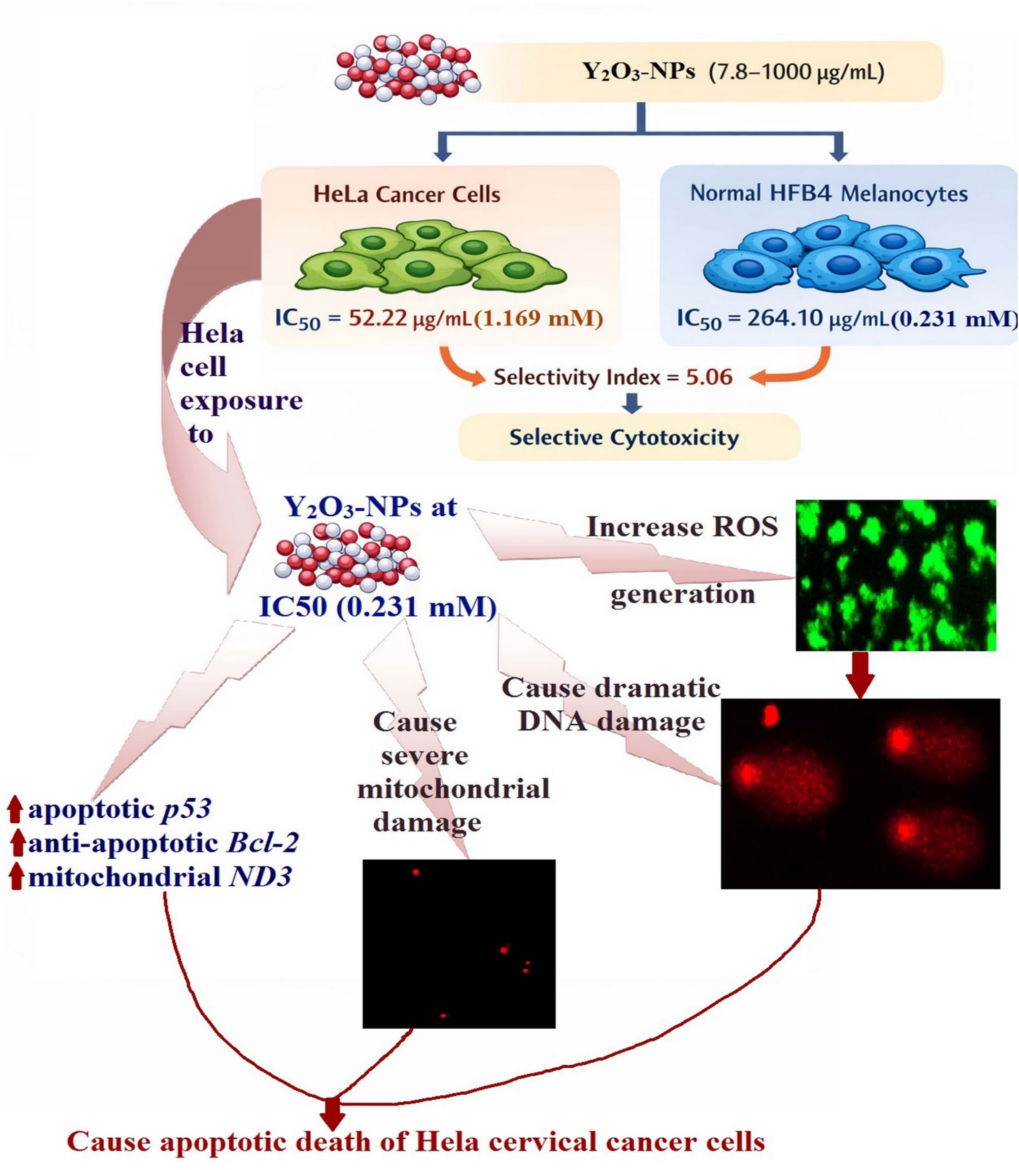
Mechanistic pathway of Y_2_O_3_-NPs-induced intrinsic mitochondrial apoptosis in human Hela cervical cancer cells. The schematic summarizes key experimental findings; ROS generation, DNA damage, mitochondrial depolarization, and apoptosis, and is interpretive; not all parameters were directly measured.

Accordingly, this study was designed to provide a systematic *in vitro* evaluation of the cytotoxic, genotoxic, oxidative, mitochondrial, and apoptosis-associated responses to Y₂O₃-NP exposure in HeLa cervical cancer cells, alongside a preliminary comparison with non-malignant human HFB4 melanocytes. The aim was to generate exploratory data on cellular stress responses rather than to establish therapeutic efficacy. The schematic presented in Fig. 7 serves as a hypothetical and integrative model that summarizes the measured endpoints and is not intended to represent a fully validated signaling pathway. By integrating viability assessment with measurements of ROS generation, DNA damage, mitochondrial membrane potential, apoptotic morphology, and selected gene expression changes, the study shows that Y₂O₃-NP exposure is associated with oxidative stress, genomic DNA damage, mitochondrial dysfunction, and apoptosis-like features in HeLa cells. These observations are consistent with activation of mitochondria-associated apoptotic processes; however, they do not constitute definitive proof of specific pathway involvement. In particular, references to *p53* signaling have been tempered to reflect *p53* transcriptional upregulation or stress association, as functional *p53* activity was not assessed and is known to be dysregulated in HPV-positive HeLa cells. Collectively, the findings should therefore be interpreted as preliminary evidence of nanoparticle-induced cellular stress responses in cervical cancer cells, forming a basis for more detailed mechanistic and translational studies.

HeLa cells were selected as the primary cellular model for this study due to their status as the definitive benchmark for HPV-driven carcinogenesis. As an immortalized line derived from an aggressive cervical adenocarcinoma, they constitutively express the HPV18 E6 and E7 oncoproteins, providing a stable platform to investigate key molecular mechanisms such as apoptosis resistance and DNA-damage repair^10–12^. The rapid proliferation and pronounced chromosomal instability inherent to HeLa cells mirror the aggressive nature of clinical cervical malignancies. Furthermore, their dysregulated redox homeostasis and heightened sensitivity to oxidative stress, biomarkers of mitochondrial dysfunction, render them an ideal substrate for evaluating the cytotoxic potential of the nanoparticles investigated here^12–14^. Meanwhile, HFB4 melanocytes were utilized as a non-malignant human cell model to enable a preliminary comparison of Y_2_O_3_-NPs cytotoxicity between cancerous and normal cells. Although HFB4 cells are not are not derived from cervical tissue and therefore do not serve as a tissue-matched cervical control, these cells offer well-documented stability in culture, reproducible growth characteristics, and have been widely used in general cytotoxicity and nanotoxicology studies^39,40^. Their inclusion provided an initial reference for safety and selectivity, allowing assessment of whether Y_2_O_3_-NPs exert differential effects on malignant versus non-malignant human cells.

In the current study, Y_2_O_3_-NPs demonstrated pronounced cytotoxic and genotoxic effects in HeLa cervical cancer cells. Importantly, these findings align with previous reports in other malignancies, where Y_2_O_3_-NPs induced significant antiproliferative effects in breast, pancreatic, hepatocellular, and epidermoid cancers^16–19^. Similar to those studies, our data indicate that oxidative stress is a central mediator of cytotoxicity. However, a key distinction of the present work lies in extending these observations to cervical cancer cells, a model that has been largely underexplored in the context of Y_2_O_3_-NPs. The observed IC50 value of 52.22 µg/mL (0.231 mM) in HeLa cells, together with the markedly high selectivity index (SI = 5.06), indicates a high sensitivity of Y_2_O_3_-NPs toward cancerous Hela cervical cells compared to normal HFB4 melanocytes, supporting a degree of cancer selectivity. This selective cytotoxicity is consistent with prior studies on metal oxide nanoparticles, where cancer cells exhibit greater susceptibility due to elevated basal ROS levels and compromised antioxidant defenses. Comparable ROS-dependent selectivity has been reported for nanoparticles such as zinc oxide, titanium dioxide, and cerium oxide nanoparticles, suggesting that redox imbalance is a broadly exploitable vulnerability in cancer cells^41,42^. In this context, Y_2_O_3_-NPs appear to follow a similar mechanistic paradigm, reinforcing their relevance within the broader field of redox-based nanotherapeutics.

Mechanistically, the present findings demonstrate a significant increase in intracellular ROS generation level following Y₂O₃-NPs exposure in cancerous Hela cervical cells. This observation is in strong agreement with previous studies on Y₂O₃-NPs in breast, hepatocellular carcinoma, epidermoid skin cancer and pancreatic cancer models, where ROS overproduction was identified as the primary upstream trigger of cytotoxicity^16–19^. Importantly, our study further links ROS generation to downstream genomic instability, as evidenced by significant DNA damage detected in Y_2_O_3_-NPs–treated HeLa cancer cells using comet assay. Quantitative analysis showed marked increases in comet parameters, including tail length, %DNA in the tail, and tail moment, reflecting extensive DNA strand breaks and genomic instability compared with untreated control Hela cells. This ROS–DNA damage axis has been widely reported in nanoparticle-mediated cancer therapy and represents a critical mechanism by which oxidative stress translates into cell death^43,44^. Collectively, these findings support the concept that Y_2_O_3_-NPs exploit the intrinsic redox imbalance of cervical cancer cells. By inducing excessive ROS accumulation, these nanoparticles trigger oxidative DNA damage, which in turn promotes selective cancer cell killing. This mechanism aligns with established redox-based anticancer strategies, highlighting the potential of Y_2_O_3_-NPs as a targeted therapeutic approach for cervical carcinoma^16–19^.

Beyond genotoxicity, mitochondrial dysfunction emerged as a central event in nanoparticle-induced cytotoxicity. The loss of Mitochondrial membrane potential is a critical hallmark of intrinsic apoptosis and reflects the inability of mitochondria to maintain their electrochemical gradient, which further enhances oxidative stress and initiates apoptotic signaling cascades^45,46^. The marked loss of mitochondrial membrane potential observed in this study is consistent with earlier reports demonstrating mitochondrial depolarization as a hallmark of Y_2_O_3_-NPs exposure in other cancer cell types^17–19^. These findings support the concept that mitochondria are primary intracellular targets of nanoparticle-induced oxidative stress, where excessive ROS disrupt mitochondrial membrane integrity, impair electron transport chain activity, and promote opening of the mitochondrial permeability transition pore, ultimately culminating in mitochondrial membrane potential collapse^47,48^. Compared with other nanomaterials, such as silver or iron oxide nanoparticles, which may induce a combination of apoptotic and necrotic cell death, Y_2_O_3_-NPs appear to preferentially activate mitochondria-associated apoptotic pathways under the present conditions^17–19^.

The induction of apoptosis by Y_2_O_3_-NPs in HeLa carcinoma cells was further confirmed by characteristic nuclear morphological changes, including chromatin condensation, nuclear fragmentation and the formation of apoptotic bodies, together with the marked increases in the percentage of apoptotic cells. These morphological alterations are classical hallmarks of programmed cell death and indicate that Y_2_O_3_-NPs–mediated cytotoxicity is not a result of random necrotic damage but rather involves highly regulated apoptotic pathways^49,50^. These findings are consistent with previous studies in non-cervical cancer models, where Y_2_O_3_-NPs triggered intrinsic apoptosis through mitochondrial disruption in human triple-negative breast cancer, hepatocellular carcinoma, epidermoid skin cancer, and pancreatic cancer cells^16–19^. Notably, the convergence of ROS generation, DNA damage, and mitochondrial depolarization observed in this study supports a coordinated mechanism of action that closely mirrors established oxidative stress–driven apoptotic pathways reported for other metal oxide nanoparticles.

At the molecular level, the observed upregulation of *p53* in Y₂O₃-NPs-treated Hela cancer cells is in agreement with studies demonstrating activation of stress-response pathways following Y₂O₃-NPs-induced DNA damage^16–19^. *p53* is a well-characterized tumor suppressor that responds to DNA damage and oxidative stress and can promote intrinsic apoptosis through transcriptional regulation of downstream pro-apoptotic factors, mitochondrial outer membrane permeabilization, and caspase activation^51,52^. However, in contrast to many cancer models where functional *p53* signaling plays a central role, the relevance of this pathway in HeLa cells is limited due to HPV-mediated *p53* inactivation. Therefore, unlike previous reports that directly link *p53* activation to apoptosis, our findings suggest a *p53*-associated stress response rather than definitive *p53*-dependent apoptosis, highlighting an important mechanistic distinction specific to cervical cancer biology.

Similarly, the upregulation of *ND3* gene expression observed in this study is consistent with reports linking mitochondrial complex I dysfunction to increased ROS production and apoptosis in nanoparticle-treated cancer cells. This supports the growing recognition of mitochondrial respiratory chain disruption as a key mediator of nanoparticle-induced cytotoxicity. Overexpression of ND3 disrupts the stability and function of complex I, impairing electron transport efficiency and promoting excessive electron leakage, which results in elevated mitochondrial ROS levels^44,53,54^. Compared with other studies, our findings further emphasize the role of ND3 as a potential contributor to mitochondrial dysfunction, although its precise mechanistic involvement requires further validation^18,19^.

Accumulated ROS induce oxidative damage to mitochondrial lipids, proteins, and DNA, triggering opening of the mitochondrial permeability transition pore and collapse of the mitochondrial membrane potential, key events in the activation of intrinsic apoptosis^39,55^. This mitochondrial depolarization facilitates the release of pro-apoptotic factors into the cytosol, leading to activation of the intrinsic, mitochondria-dependent apoptotic pathway. Therefore, marked *ND3* overexpression reflects severe mitochondrial stress and functional impairment, highlighting mitochondria as a primary target of Y_2_O_3_-NPs–mediated cytotoxicity Hela cancer cells. Furthermore, these findings suggest that *ND*3 functions not only as a sensitive marker of mitochondrial perturbation but also as a critical molecular mediator and potential therapeutic target for nanoparticle-induced apoptosis, linking mitochondrial dysfunction, ROS generation, and activation of the intrinsic apoptotic cascade^56–59^.

Interestingly, the increased expression of *Bcl-2* observed in this study contrasts with the expected downregulation typically associated with apoptosis induction. *Bcl-2* is traditionally known for its anti-apoptotic role in stabilizing mitochondrial membranes and inhibiting cytochrome c release^57–59^. However, similar paradoxical increases in *Bcl-2* expression have been reported under conditions of severe oxidative stress and mitochondrial dysfunction, where compensatory cellular responses are activated but ultimately fail to prevent apoptosis^60–62^. Persistent mitochondrial injury and continued ROS accumulation can overwhelm *Bcl-2*–mediated protective effects, allowing intrinsic apoptotic pathways to proceed^49,60^. Therefore, the observed *Bcl-2* marked upregulation is interpreted as a possible compensatory response that is insufficient to counteract apoptosis under Y₂O₃-NPs–induced persistent stress, consistent with previous nanoparticle studies demonstrating that oxidative damage can override anti-apoptotic defenses and drive programmed cell death despite adaptive responses.

Taken together, these findings position Y_2_O_3_-NPs within the broader class of ROS-generating nanotherapeutics, with a mechanism of action that is largely consistent with other metal oxide nanoparticles but with specific distinctions related to cervical cancer biology. The observed combination of selective cytotoxicity, ROS-mediated DNA damage, mitochondrial dysfunction, and apoptosis highlights a multifaceted mechanism that is comparable to, yet distinct from, previously reported nanoparticle systems. Importantly, this study contributes to the field by providing the first integrated analysis of these mechanisms in cervical cancer cells, addressing a notable gap in the literature. However, consistent with prior nanoparticles studies, the present work remain limited to *in vitro* conditions and require further validation in more complex biological systems. Future investigations should focus on comparing Y_2_O_3_-NPs with established chemotherapeutic agents and other nanomaterials under standardized conditions to better define their relative efficacy and translational potential. Cell viability in this study was assessed using the MTT assay, a widely validated metabolic method, although it can be influenced by mitochondrial activity. To strengthen these findings, cytotoxic effects were corroborated by complementary endpoints, including ROS generation, DNA damage, and apoptosis markers. Nevertheless, future studies employing orthogonal viability assays (e.g., crystal violet staining, LDH release) would further confirm and extend these observations.

Additional limitations include the use of a single cervical cancer cell line (HeLa) and one normal cell line (HFB4 melanocytes), which may not fully capture the biological heterogeneity of cervical cancers or normal tissues. Experiments were exclusively *in vitro*, and therefore in vivo studies are required to evaluate biodistribution, systemic toxicity, pharmacokinetics, and therapeutic efficacy. Furthermore, mechanisms of nanoparticle internalization, long-term cellular responses, and potential off-target effects were not investigated and warrant further study. Despite these limitations, the present work provides a strong mechanistic foundation supporting the potential of Y_2_O_3_-NPs as selective nanotherapeutic agents for cervical cancer. These results underscore the importance of further preclinical studies to assess tissue-specific effects and advance toward potential clinical applications.

## Conclusion

This study provides preliminary evidence that Y_2_O_3_-NPs markedly reduce HeLa cervical cancer cell viability in a concentration-dependent manner, while exerting comparatively lower cytotoxic effects on normal HFB4 melanocytes under the tested conditions. Treatment of HeLa cells with Y_2_O_3_-NPs at the IC50 concentration was associated with increased ROS generation, significant genomic DNA damage, marked mitochondrial membrane depolarization, and pronounced apoptotic nuclear changes. These results suggest that the cytotoxic effects of Y_2_O_3_-NPs may involve oxidative stress and mitochondria-associated apoptotic pathways, although the precise molecular mechanisms remain to be fully elucidated. Quantitative analyses consistently showed that HeLa cells were more affected than HFB4 melanocytes, indicating a potential preferential effect on cancer cells. However, as this study was limited to a single cervical cancer cell line and one normal cell type, the findings should be considered exploratory and not broadly generalizable. Overall, the data indicate that Y_2_O_3_-NPs can induce cellular stress responses that link ROS accumulation, DNA damage, and mitochondrial dysfunction to apoptosis in HeLa cells. These findings provide a basis for further preclinical research, including studies involving additional cancer and normal cell models, in vivo experiments, and more detailed molecular analyses, to confirm efficacy, selectivity, and safety. In summary, this study establishes an initial mechanistic framework demonstrating that Y_2_O_3_-NPs can exert potent cytotoxic effects on HeLa cervical cancer cells, supporting their potential as preclinical nanotherapeutic candidates, while highlighting the need for further investigation to validate these preliminary findings and fully characterize their therapeutic potential.

## Data Availability

The datasets used and/or analyzed during the current study are available from the corresponding author on reasonable request.
